# Research models of neurodevelopmental disorders: The right model in the right place

**DOI:** 10.3389/fnins.2022.1031075

**Published:** 2022-10-20

**Authors:** Eleni Damianidou, Lidia Mouratidou, Christina Kyrousi

**Affiliations:** ^1^University Mental Health, Neurosciences and Precision Medicine Research Institute “Costas Stefanis”, Athens, Greece; ^2^First Department of Psychiatry, Medical School, Eginition Hospital, National and Kapodistrian University of Athens, Athens, Greece

**Keywords:** neurodevelopmental disorders, malformations of cortical development, disease modeling, animal models, two-dimensional (2D) human-specific cultures, brain organoids

## Abstract

Neurodevelopmental disorders (NDDs) are a heterogeneous group of impairments that affect the development of the central nervous system leading to abnormal brain function. NDDs affect a great percentage of the population worldwide, imposing a high societal and economic burden and thus, interest in this field has widely grown in recent years. Nevertheless, the complexity of human brain development and function as well as the limitations regarding human tissue usage make their modeling challenging. Animal models play a central role in the investigation of the implicated molecular and cellular mechanisms, however many of them display key differences regarding human phenotype and in many cases, they partially or completely fail to recapitulate them. Although *in vitro* two-dimensional (2D) human-specific models have been highly used to address some of these limitations, they lack crucial features such as complexity and heterogeneity. In this review, we will discuss the advantages, limitations and future applications of *in vivo* and *in vitro* models that are used today to model NDDs. Additionally, we will describe the recent development of 3-dimensional brain (3D) organoids which offer a promising approach as human-specific *in vitro* models to decipher these complex disorders.

## Introduction

The cerebral cortex is responsible for many of the higher-level cognitive functions in humans, such as perception, decision making, and language. Disruption of the tightly coordinated processes regulating brain development provokes the so-called neurodevelopmental disorders (NDDs), characterized by cognitive deficits, developmental delay and intellectual disabilities (ID). Amongst the most common NDDs are autism spectrum disorder (ASD) and attention deficit and hyperactivity disorder (ADHD) (Doernberg and Hollander, 2016). A group of diseases caused by defective cortical formation, namely malformations of cortical development (MCDs), belong also to the complex collection of NDDs (Desikan and Barkovich, 2016), which are usually present in patients with ASD and ADHD concurrently (Ho et al., 2019). Defining the pathophysiological mechanisms which underlie brain developmental disorders will help to facilitate treatment in NDDs, while it will contribute to the limited knowledge we have on human brain development. For this reason, in recent years, scientists have made a significant effort to model such disorders.

**FIGURE 1:**
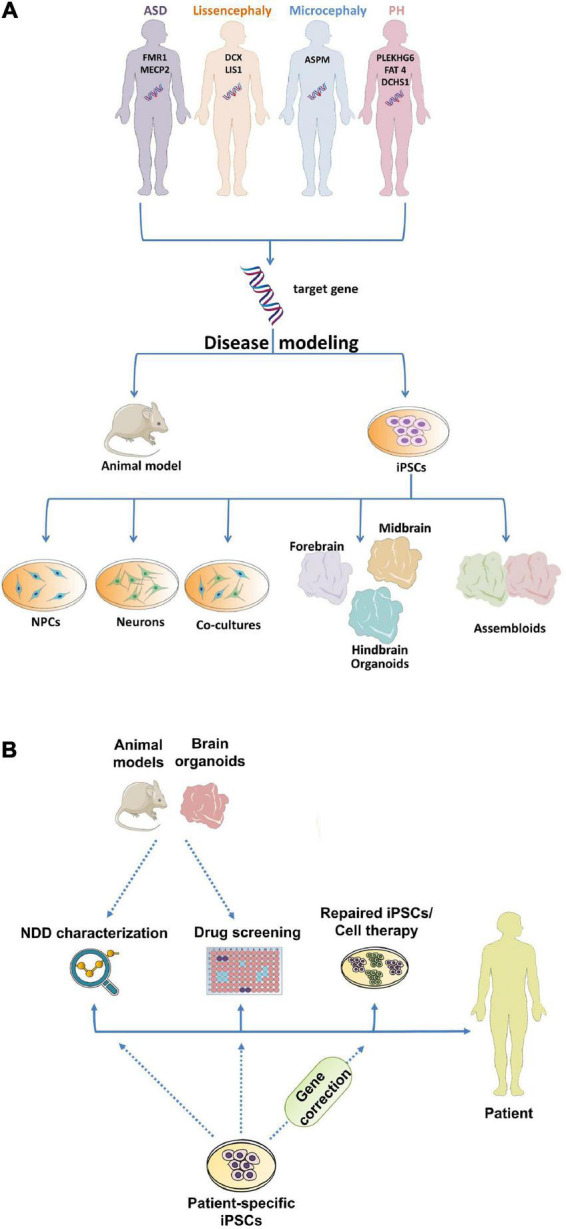
Utilization of different model systems and approaches in neurodevelopmental research. **(A)** Modeling of NDDs using *in vivo* and *in vitro* model systems. **(B)** Human-specific iPSC model systems are used for NDD characterization, drug screening and stem cell-based therapies, whereas animal models and human-specific COs can be used for NDD characterization and drug screening, aiming to improve patients’ quality of life. ASD, Autism Spectrum Disorder; PH, Periventricular Heterotopia; iPSCs, induced pluripotent stem cells; NPCs, neural progenitor cells; NDD, Neurodevelopmental disorder.

Until recently, animal models played a central role in the investigation of NDDs, however, they cannot fully recapitulate the human clinical and/or molecular and cellular phenotypes (Zhao and Bhattacharyya, 2018). Considering that there is limited access to fetal human brain tissue due to ethical and practical constraints, the use of alternative human-specific model systems that are accessible and ethically justified is crucial. In this review, we will introduce the basic steps of brain development and the evolutionary differences among mammals. The advantages and limitations of the *in vivo* and *in vitro* models for NDDs will be also analyzed. Finally, we will review the key categories of NDDs and MCDs and the models used to investigate the implicated mechanisms (Figures 1A,B).

## Evolutionary perspectives of mammalian neurogenesis

The development of the cerebral cortex begins early in the first trimester of gestation when neuroepithelial cells (NEs), the founder neural progenitor population located in the most rostral part of the neural tube, divide symmetrically to expand the neuroepithelial area (Subramanian et al., 2017). NEs, which form the ventricular zone (VZ) of the developing cortex, are polarized cells extending two thin processes: one contacting the apical side and the other contacting the basal side of the developing brain (pial) (Villalba et al., 2021). Shortly after the onset of corticogenesis, they transform into apical radial glia cells (aRGs). aRGs divide symmetrically, expanding the progenitor pool, and asymmetrically, giving rise to neurons directly or indirectly through generating other progenitor cells (Noctor et al., 2004). Amongst these progenitors are the intermediate progenitors (IPs) and the basal radial glia cells (bRGs) colonizing the subventricular zone (SVZ) and outer SVZ (oSVZ), respectively (Kalebic and Huttner, 2020). After their generation, newborn neurons assume a multipolar morphology and via the guidance of the RGs’ basal processes migrate away from the VZ and SVZ toward the developing cortical plate (CP) (Villalba et al., 2021). The continuous neuronal generation leads to the sequential formation of cortical layers (L1-L6) in an inside-out manner, where deep layer neurons form L5 and L6 and upper layer neurons form L2, L3, and L4 (Franchini, 2021).

Even though the main steps of neurogenesis are common among mammals (Franchini, 2021), the human cortex is greatly expanded, which is thought to be the foundation for the unique intellectual abilities of humans. A hallmark of humans’ but also of other primates’ and gyrencephalic cortices is the enlargement of supragranular layers, which originate from a unique progenitor pool, the oSVZ progenitors (Smart et al., 2002). In gyrencephalic species, bRGs are the most abundant neural progenitor (NPC) population in the SVZ (Hansen et al., 2010), and at the cellular level, they are highly heterogenous: there are bRGs with basal but no apical process, bRGs with only an apical process or bRGs with both basal and apical process (Betizeau et al., 2013). On the contrary, bRG-like cells in lissencephalic species like mice are few in number (5–10% of total NPCs between E12-E18) presenting only a basal process and located in the upper part of the SVZ (Wang et al., 2011). In gyrencephalic species, bRGs present a high proliferative capacity, whereas in mice they are mostly neurogenic (Smart et al., 2002; Betizeau et al., 2013). Thus, in gyrencephalic species and in particular, in humans, oSVZ is thicker with its progenitor cells playing a significant role in the formation of cortical folds (Borrell and Götz, 2014). Mechanical forces have highly contributed to the emergence of a folded cortex, with intercellular adhesion of migrating neurons being a crucial factor underpinning cortical folding (del Toro et al., 2017). Additionally, one of the main elements influencing cortical folding is the extracellular matrix (ECM) with human ECM components defining the mechanical properties of the developing cortex (Amin and Borrell, 2020). Transcriptional analysis in the mouse and primate brains has led to the identification of primate-specific and human-specific genes regulating the expansion of the neocortex (Boroviak et al., 2018). Characteristic examples of such human-specific genes are *NOTCH2NL* which contributes to the rapid evolution of the expanded human cortex (Fiddes et al., 2018) and *ARHGAP11B* which increases its size and folding (Florio et al., 2015). Primate-specific and bRG-specific genes were uncovered in fetal human brain samples, such as *TMEM14B, DAG1, KCNK10, HP1BP3* (Liu et al., 2017). Also, the hominin-specific gene *TBC1D3* was found to increase ERK signaling in humans’ bRGs and therefore, their proliferative capacity (Ju et al., 2016). A recent study by Pinson et al. revealed that a single lysine-to-arginine substitution in human TKTL1 leads to increased bRGs number and greater neurogenesis than in Neanderthals (Pinson et al., 2022). Nevertheless, the newly identified human-specific genes or human-specific isoforms cannot explain the phenotypic and morphological differences that are observed in human evolution as mentioned above, thus, attention has also been drawn to non-coding and regulatory sequences (Liu et al., 2017). Comparative genomic and epigenomic studies have identified: (a) regions in the human lineage named human accelerated regions (HARs), (b) non-coding region changes enriched in developmental enhancers (Bird et al., 2007) and (c) regions named human gained enhancers (HGEs) (Vermunt et al., 2016). These regions regulate gene expression of crucial key neurodevelopmental genes, some of which are associated with patterning in the frontal cortex, such as *TBR1* (Won et al., 2019). Beyond these, other genes were proposed to be associated with the size of the frontal cortex such as *FGF17* and *EMX2* (Cholfin and Rubenstein, 2008). All the above, eventually lead to an increase in the neuronal number accompanied by a non-analog increase in the brain area size, which in turn leads to cortical gyrification. Interestingly, variants in human-specific genes, genes with human-specific expression pattern or HARs and HGEs have been found enriched in patients with NDDs, suggesting that human-specific changes are important not only for the evolution of the human brain but also for brain-related disorders.

## Model systems: Advantages and disadvantages in studying brain development and disease

### *In vivo* models

Animal models have been used for many years to dissect the mechanisms and disease-causing factors implicated in NDDs (Figures 1A,B), providing a huge advantage over human tissues whose use is limited. Among the animal models that have been highly used for studying brain development and brain-related diseases are zebrafish (*Danio rerio*) (Howe et al., 2013) and fruit fly (*Drosophila melanogaster*) (Gatto and Broadie, 2011). Their short life cycle and high genomic similarity with humans make them ideal to screen multiple candidate genes relatively fast. Drosophila contains almost 87% of genes involved in neurological and 75% of genes implicated in neurodevelopmental function. Similarly, zebrafish shares almost 70% homology with human genes with its neurodevelopmental processes being highly conserved through evolution (Howe et al., 2013). The chicken embryo (*Gallus gallus*) has also been a valuable tool in vertebrate neurodevelopment due to its large size, fast growth, easy accessibility for visualization and experimental manipulation and cost-effectiveness (Vergara and Canto-Soler, 2012; Zosen et al., 2021). Even though a huge breakthrough has been made in the research of brain-related disorders using these animal models, it is definite that they are distant from the human evolutionary perspective. Toward bridging this gap, scientists have turned to other model systems, such as rodents, to decipher the molecular and cellular mechanisms governing NDDs. The mouse model has been widely used because it presents a relatively fast reproduction time with several offspring. In addition, it presents more than 95% similarity with the human genome allowing accurate genomic manipulation of candidate genes. However, the use of mice also presents some limitations as it lacks several human-specific features, such as the gyrification of the cortex (Boroviak et al., 2018). Behavioral tests in rodents have been performed to model human behavioral deficits in individuals with NDDs (Kazdoba et al., 2016). Mice models exhibit several human NDD-related behavioral deficits including cognitive impairments, defects in learning and memory, hyperactivity and autism-like behaviors, but others cannot be precisely recapitulated, suggesting that assessment of higher brain functions in the lissencephalic brain is challenging. To overcome these limitations, non-human primates (NHPs) were proposed as potential models. The most used NHP is the monkey (*Rhesus macaque*), which presents a plethora of similarities with the human brain regarding cognitive function but also in terms of anatomical, structure, molecular and cellular features (Liu et al., 2020). Due to their long reproductive rate and demands in hosting they have only recently started to be used in NDDs research (Feng et al., 2020). Nevertheless, with the advantages of *in vivo* transient gene manipulation—i.e., *in utero* electroporation—and CRISPR/Cas9 technology precise nucleotides changes and transgene insertions/deletions—it has become economical and time effective to generate genetically manipulated NHPs (Chen et al., 2017; Zuo et al., 2017; Heide et al., 2020). These animal models serve as significant research tools to study genetic aspects of NDDs manifestation with great translational potentials.

### Human-specific *in vitro* model systems

The striking differences between animal models and humans in terms of brain development raise questions about the value of information gained from animal studies. Considering that the use of human embryos or fetuses is ethically restricted, human-specific *in vitro* models could be a complementary approach to animal studies. A major breakthrough has been the differentiation of human embryonic stem cells (hESCs) and human induced pluripotent stem cells (hiPSCs) into all different cell lineages, possibly including neuronal lineage (Ardhanareeswaran et al., 2017). With the use of different culture media, hESCs and hiPSCs have been differentiated in two-dimensional (2D) neuronal cultures (Park et al., 2008), namely the monolayer cell cultures, including dopaminergic neurons (Sánchez-Danés et al., 2012), GABAergic neurons (Yang et al., 2017), serotonergic neurons (Vadodaria et al., 2016), hippocampal dentate granule neurons (Yu et al., 2014), motor neurons (Park et al., 2016), and hypothalamic neurons (Merkle et al., 2015). Besides neuronal cultures, astrocytes (Juopperi et al., 2012), oligodendrocytes, and microglia (Abud et al., 2017) have also been generated. These cultures have been used to study neuronal differentiation, neuronal activity, and neuronal morphology. The co-culture of neuronal and glial cells was also implemented to generate a more complex system and study cell-to-cell communication (Figure 1A). Considering that iPSCs enable the generation of scalable quantities of patient tissues, they facilitate the identification of therapeutic targets through drug screening (Elitt et al., 2018; Figure 1B). Moreover, aside from their contribution in NDD characterization and drug testing, iPSCs potential lies in their ability to serve as initial material for patient-specific stem cell-based cell therapies (Madrid et al., 2021; Figure 1B). Even though 2D human cultures transcended animal models concerning studying species-specific mechanisms, limitations arise from their use, especially regarding their inability to recapitulate cellular homeostasis and diversity. In particular, the lack of cellular diversity and cell interconnection among different brain regions, even in the co-culture system, may lead to the underestimation of the implicated cellular and molecular mechanisms.

Given these restrictions, the use of three-dimensional (3D) cell cultures, namely brain organoids which have been developed in the last decade, is pioneering in the study of brain development and disease. Brain organoids are stem cell-derived self-organized 3D tissues that mimic the cytoarchitecture, cellular composition and gene expression profile of the fetal human brain. The principle of their generation is based on cell sorting and spatially restricted lineage commitment (Benito-Kwiecinski and Lancaster, 2020). This model system is characterized by a high cellular diversity, where different NPCs, newborn and mature neurons as well as glial cells form a highly orchestrated and organized structure. Gene expression and electrophysiological studies have shown that brain organoids accurately resemble the first and second trimesters of the human fetal brain (Watanabe et al., 2017; Qian et al., 2018). In addition, single-cell RNA-sequencing data suggest that they model precisely cortical development on the molecular level (Camp et al., 2015; Amiri et al., 2018; Kanton et al., 2019; Velasco et al., 2019). Cerebral organoids (COs), the first brain organoid protocol (Lancaster et al., 2013; Lancaster and Knoblich, 2014), were generated from iPSCs based on their intrinsic properties to generate neuroectoderm. This protocol has been used since then to mimic early human brain development and evolution, as well as human brain diseases (Kyrousi and Cappello, 2020). Areas with specific regional characteristics like dorsal and ventral forebrain, choroid plexus, hippocampus and retina were found within these organoids (Lancaster et al., 2013). Interestingly, these regions can form connections with each other, making the modeling of interconnectivity possible. Newer protocols have attempted to direct regional identity in a more guided way by using patterning factors (Qian et al., 2019). Examples of single-region brain organoids include organoids of the cortex (Velasco et al., 2019), hypothalamus (Qian et al., 2016; Huang et al., 2021), cerebellum (Silva et al., 2021), and midbrain (Qian et al., 2016; Galet et al., 2020). In addition, protocols generating fused patterned organoids (or assembloids) have been developed to recapitulate the connections of different brain regions (Figure 1A). The fusion of dorsally and ventrally patterned organoids made possible to model the migration of inhibitory and excitatory neurons between and within these two areas, as well as the formation of a local inhibitory–excitatory neuronal circuit (Bagley et al., 2017; Birey et al., 2017). The generation of cortico-striatal assembloids (Miura et al., 2020) to model human cortico-striatal circuits of the forebrain has also been a big achievement, as dysfunctions in neural circuits of this pathway are thought to contribute to NDDs. The co-culture of medial ganglionic eminence organoids (MGEOs) and cortical organoids, generating human fused MGE-cortical organoids (Xiang et al., 2017) has been established to investigate complex interaction between specific brain regions. Following this approach, the fusion of human thalamic and COs to model axon projections (Xiang et al., 2019) was developed to study the molecular basis of human complex activities such as sensory-motor processing and attention. The different organoid protocols were shown to recapitulate closer the key aspects of human brain development than other model systems. Overall, the new methodologies in stem cell technology have expanded our toolbox for modeling NDDs. Choosing the appropriate model system depends on the focus of the investigation and the research question that needs to be answered (Figures 1A,B).

## Using the correct system in modeling neurodevelopmental disorders

Neurodevelopmental disorders comprise a heterogeneous group of disorders characterized by a plethora of clinical phenotypes such as cognitive impairment, communication deficits, impaired psychomotor skills, and inability to reach developmental milestones (Parenti et al., 2020). NDDs include ASD, ADHD, ID, communication disorders, neurodevelopmental motor, learning and speech disorders. This group of disorders is characterized by high rates of comorbidity between several diseases within this diagnostic group. For example, individuals with ASD and ID exhibit often MCDs (Garcia-Forn et al., 2020), a wide spectrum of cortical abnormalities. MCDs are classified based on the perturbed developmental processes, thus, alterations in proliferation lead to microcephaly and macrocephaly, defects in neuronal migration result in periventricular/subcortical band heterotopia and Lissencephaly and deficits in the cortical organization lead to polymicrogyria (Barkovich et al., 2012; Severino et al., 2020). MCDs clinical manifestations include epilepsy, autistic features, ID and developmental delay, coupling MCDs with neuropsychiatric disorders. For example, individuals with Rett Syndrome present microcephaly and ASD at the same time, while in Seckel and Angelman syndrome patients exhibit microcephaly and ID (Sisodia et al., 2014). Patients with Prader-Willi (Bennett et al., 2015) and Timothy syndrome (Bauer et al., 2021) suffer from both ID and ASD, while in Fragile X syndrome characteristics of ASD, ID, and ADHD are present simultaneously. Interestingly, while monogenic causes predominate in MCDs (Table 1), NDDs usually exhibit polygenic pathophysiology (Li M. et al., 2018). Nevertheless, the etiology of these different syndromes is not well-understood up till now and thus, tremendous effort has been made to scrutinize their causality by using different model systems which will be presented in the following sections (Figures 1A,B). In addition, we will present MCDs based upon the observed neuropathological phenotype: (i) presence of ectopic neurons (periventricular heterotopia and subcortical band heterotopia), (ii) change of the gyrification index (Lissencephaly and polymicrogyria), (iii) abnormal brain size (microcephaly and macrocephaly) and how can we model them using the aforementioned model systems.

**TABLE 1:** Animal and human-specific model systems used in NDDs research.

Pathophysiology	Mutated gene/Chemical exposure	Phenotype in animal model	Phenotype in human *in vitro* model
FXS, ASD	*FMR1*	***Fmr1*-KO mouse**: Disrupted dendrite morphology and neurotransmission, lack of robust cognitive impairments (Kazdoba et al., 2014)	**3D forebrain organoids**: Reduced NPCs’ proliferation, increased synapse formation and neuronal hyperexcitability (Kang et al., 2021)

RTT, ASD	*MECP2*	***Mecp2* deficient mouse**: Cognitive deficits, abnormalities in locomotion, and stereotypes (Stearns et al., 2007)	**2D neuronal cultures**: Fewer synapses, smaller soma size, and disrupted firing activity (Marchetto et al., 2010)
		
		***Mecp2* conditional KO mouse**: Severe neurological symptoms (Guy et al., 2001)	**3D MGEOs and cortical organoids**: Deficits in forebrain development, interneuron differentiation, neuron fate commitment, and synaptic transmission (Xiang et al., 2020)
		
		***Mecp2* mutant mouse**: Impaired learning and memory (Moretti et al., 2006)	**3D COs**: Lateral expansion of ventricles, enhanced NPCs’ proliferation, impaired neurogenesis, and neuronal migration (Mellios et al., 2018)
		
		***Mecp2* null mouse**: Metabolic disturbances (Goffin and Zhou, 2012)	**3D DFOs and VFOs**: Neuronal functional defects and disrupted interneuron migration (Gomes et al., 2020)

Tuberous sclerosis, ASD	*TSC1/2*	***Tsc1* null mouse**: Embryonic death at mid-gestation (Kwiatkowski et al., 2002)	**2D neuronal cultures**: Disrupted synaptic transmission (Costa et al., 2016), enlargement of soma, disrupted neurite outgrowth, and impaired intercellular connection (Martin et al., 2020)
		
		***Tsc1* conditional KO mouse**: Enlarged ectopic neurons, reduced myelination, presence of seizures, and limited survival (Meikle et al., 2007)	**2D NPCs**: Enlarged cell size, enhanced proliferation, altered neurite outgrowth (Martin et al., 2020), and delayed neuronal differentiation (Zucco et al., 2018)
		
		***Tsc1*^+/–^ mouse**: Impaired learning and social behavior. Absence of cerebral lesions and spontaneous seizures (Goorden et al., 2007)	**2D pNSCs**: Increased proliferation (Li et al., 2017b)
		
		***Tsc2*^+/–^ mouse**: Deficits in memory and learning. Absence of neuropathology and seizures (Ehninger et al., 2008)	**2D *TSC2* deficient neuronal cultures**: Enlarged cell body size, increased process outgrowth, and mTORC1 hyperactivation (Winden et al., 2019)
		
			**3D COs**: Presence of tumors and cortical tuber lesions. Identification of CLIP cells in this model system (Eichmüller et al., 2022)

ADHD	Genes of the GRM family, *PARK2, SLC6A3*, exposure to BPA and phthalates	**DAT-KO mouse**: Hyperactivity, sleep dysregulation, and cognitive deficits (Kasahara et al., 2013)	**3D forebrain organoids treated with BPA**: Dose-dependent decrease in the VZ thickness and reduction of EdU^+^ and PH3^+^ NPCs (Qian et al., 2016)
		
		**DAT-KO rat**: Defects in working memory and behavioral alterations affecting reward processing and decision making (Cinque et al., 2018)	

PH	*FLNA, MAP1B, EML1, FAT4, DCHS1, MOB2, NEDD4L, ARFGEF2, INTS8, GNG5, PLEKHG6, ECE2, LGAL3BP*	***Flna*-KD rat**: Presence of ectopic nodules lining the ventricles, impaired neuronal migration, and disrupted radial glial scaffold (Carabalona et al., 2012)	**3D *FAT4* and *DCHS1* mutant COs**: Altered NPCs’ morphology, disrupted neuronal migration, and abnormal gene expression profile in ectopic neurons (Klaus et al., 2019)
		
		**PH ferret model**: Presence of neuronal nodules in the cortex and disrupted radial glial fibers (Matsumoto et al., 2017)	**3D COs overexpressing *GNG5:*** NPCs’ delamination and migration defects (Ayo-Martin et al., 2020)
		
		**HeCo mouse**: Presence of heterotopic neurons and misplaced apical progenitors, impaired primary cilia of aRGs, and Golgi apparatus defects (Kielar et al., 2014; Bizzotto et al., 2017; Uzquiano et al., 2019)	**3D COs overexpressing *PLEKHG6***: Presence of ectopic neurons at the ventricular surface and disruption of the neuroepithelial lining (O’Neill et al., 2018b)
		
		***Fat4*-KD/*Dchs1*-KD mouse**: Abnormal distribution and increased proliferation of NPCs, disrupted neuronal differentiation (Cappello et al., 2013)	
		
		***Mob2*-KD mouse**: Altered neuronal distribution, perturbed neuronal ciliary number and position (O’Neill et al., 2018a)	**3D *ECE2* deficient COs**: Cell fate changes and accumulation of neurons in the VZ (Buchsbaum et al., 2020)
		
		***Nedd4l* mutant mouse**: Altered neurogenesis, disrupted neuronal positioning, and terminal translocation (Broix et al., 2016)	**3D *LGALS3BP* mutant COs**: Thick apical belts, decreased length of RGs’ primary cilia and mislocalized neurons (Kyrousi et al., 2021)

SBH	*LIS1, DCX, EML1*	***Dcx*-KD rat**: Presence of unilateral SBH (Lapray et al., 2010)	**2D *EML1* mutant NPCs**: Impaired primary cilia formation and disturbed function of Golgi apparatus (Uzquiano et al., 2019)
		
		**HeCo mouse**: M isplaced RGs, perturbed NPCs’ microtubule dynamics, abnormal ciliary length, and altered Golgi apparatus function of RGs (Kielar et al., 2014; Bizzotto et al., 2017; Uzquiano et al., 2019)	**3D *EML1* deficient COs**: Presence of ectopic neural rosettes and heterotopic neuronal clusters, altered ciliary length, and spindle orientation of RGs (Jabali et al., 2022)

Lissencephaly	*LIS1, DCX, ARX, RELN, KIF2A, KIF5C, CDK5, VLDLR, ACTB*, ACTG1, *TUBG1*	***Lis1* deficient mouse**: Severe cortical layering defects (Youn et al., 2009), reduced migration speed, elongated neurite lengths, disrupted synaptic physiology (Fleck et al., 2000), impairments of motor coordination and cognition (Paylor et al., 1999)	**2D *DCX* mutant NSCs**: Perturbed migration, delayed differentiation, and impaired neurite formation (Shahsavani et al., 2018)
		
		***Ndel1* mutant mouse**: Disorganization of cortical layer formation and hippocampal defects (Youn et al., 2009)	**3D MDS COs**: Increased RGs apoptosis and horizontal divisions, defective neuronal migration and cytokinesis delay of bRG-like cells (Bershteyn et al., 2017)
		
		***Dcx* mutant mouse**: Disrupted lamination of the hippocampus and defects in learning with normal neurogenesis and neuronal migration (Corbo et al., 2002)	**3D MDS forebrain organoids**: Reduced size and expansion rate, increased neurogenesis, and disrupted VZ niche architecture (Iefremova et al., 2017)

PMG	*TUBA1A, TUBB2B, TUBB3, GPR56, WDR62, EOMES, SCN3A*	***Tubb2b* mutant mouse**: Cortical thinning and increased neuronal apoptosis (Stottmann et al., 2013)	**2D primary neuronal cultures overexpressing *SCN3A***: Impaired neurite branching (Smith et al., 2018)
		
		***Gpr56* deficient mouse**: Ectopic neurons and disrupted pial basement membrane (Li et al., 2008)	**3D *EML1* deficient COs**: Upregulation of bRGs markers and ECM components (Jabali et al., 2022)
		
		***Scn3a* mutant ferret model**: Disrupted gyral formation and presence of cortical gray matter heterotopia (Smith et al., 2018)	**3D *WDR62* mutant COs**: Disruption of bRGs (Zhang et al., 2019)

Microcephaly	*ASPM, FANCD2, CDK5RAP2, CPAP, WDR62, KNL1*, ZIKV infection	***Aspm*-KD zebrafish**: Reduced head size possibly due to mitotic arrest during early development (Kim et al., 2011)	**2D *CPAP* mutant NPCs**: Reduced proliferation, elongated primary cilia, retarded ciliary disassembly, and premature neuronal differentiation (Gabriel et al., 2016)
		
		***Fancd2* deficient zebrafish**: Microcephalic phenotype and p53-dependent apoptosis (Liu et al., 2003)	**2D *KNL1* deficient NPCs**: Reduced cell growth, high cell death, and premature differentiation (Omer Javed et al., 2018)
		
		**Mouse models infected with ZIKV**: Present microcephaly (Li et al., 2016; Shao et al., 2016; Gorman et al., 2018; Julander et al., 2018; Li C. et al., 2018; Szaba et al., 2018)	**3D *CDK5RAP2* mutant COs**: Small size, decreased number of NPCs, change of NPCs’ spindle orientation, and premature neuronal differentiation (Lancaster et al., 2013)
		
		***Wdr62* mutant mouse**: Reduced brain size, decreased number of NPCs, retarded cilium disassembly, elongated cilia, and delayed cell cycle progression (Zhang et al., 2019)	**3D *ASPM* deficient COs**: Reduced size, decreased proliferation, and disorganization of NPCs (Li et al., 2017a)
		
		***Cpap* mutant mouse**: Robust apoptosis, cilia loss, RGs mislocalization, and robust heterotopia (Lin et al., 2020)	**3D *CPAP* mutant COs**: Small size, disorganized VZ-like regions, premature neuronal differentiation due to premature switch of NPCs’ cleavage plane, increased number and elongated cilia (Gabriel et al., 2016; An et al., 2022)
		
		***Aspm-*KO ferret model**: Severe microcephaly and significant reduction of cortical surface area (Johnson et al., 2018)	**3D *WDR62* mutant COs**: Reduced size, decreased NPCs’ proliferation, premature neuronal differentiation, impaired ciliary disassembly, and elongated cilia (Zhang et al., 2019)
		
		**Macaque models infected with ZIKV**: Recapitulate key features of CZS (Dudley et al., 2019)	**3D COs infected with ZIKV**: Reduction of proliferative zones (Cugola et al., 2016)
		
Macrocephaly	*RAB39B, PTEN, AKT3*	***Rab39b*-KO mouse**: Macrocephalic phenotype, impaired cortical neurogenesis, and ASD-like behavior (Zhang et al., 2020)	**3D *RAB39B* mutant COs**: Enlarged size, NPCs’ over-proliferation, and impaired neuronal differentiation (Zhang et al., 2020)
		
		***Pten* deficient mouse**: Regional brain overgrowth with cortical cultures displaying increased proliferation of glial cells (Clipperton-Allen et al., 2019)	**3D *PTEN* deficient COs**: NPCs cell cycle re-entry and over-proliferation as well as delayed neuronal differentiation leading to larger and folded organoids (Li et al., 2017c)
		
		***Akt3* mutant mouse**: Brain enlargement and low seizure threshold (Tokuda et al., 2011)	

Summary of the causative genes of the NDDs discussed in this review and the impact of their disruption in animal models and 2D/3D human-specific model systems.

### Neurodevelopmental disorders

#### Autism spectrum disorders

Autism spectrum disorders involves deficits in social communication and repetitive behavior while it presents a complex mode of inheritance concerning genetic loci on several chromosomes (Muhle et al., 2004; Marco and Skuse, 2006; Rylaarsdam and Guemez-Gamboa, 2019). Candidate genes for ASD have key roles in neurodevelopment or/and neurotransmission (Rubenstein and Merzenich, 2003; McDougle et al., 2005). These genes encode regulatory proteins, such as transcription factors involved in neuronal synapses (Zoghbi, 2003) and networks relevant to neuroinflammation and neurotransmission (Voineagu et al., 2011). ASD remains one of the most heterogeneous NDD with more than 800 associated genes (Yin and Schaaf, 2017). Some of the most well-studied monogenic syndromes correlated with ASD in humans are: Fragile X syndrome (FXS) (mutation in *FMR1*) (Sitzmann et al., 2018), Rett syndrome (RTT) (mutation in *MECP2*) (Meloni et al., 2000), and tuberous sclerosis (mutations in *TSC1* and *TSC2*) (Bjornsson et al., 1996), which will be analyzed in the next paragraphs.

The use of animal models has made and continues to make enormous progress in the understanding of the molecular mechanisms underlying FXS. The *Fmr1* knockout (KO) mouse model exhibited synaptic deficits, abnormal dendritic spine morphology and neurotransmission defects, providing opportunities to evaluate novel drug targets (Kazdoba et al., 2014). Although, studies in FXS mouse models revealed deficient metabotropic pathway (Guo et al., 2016), its modulation led to unsuccessful clinical trials. The lack of effective clinical trials so far can be attributed to the fundamental developmental, biochemical, and physiological differences between animal models and humans. Indeed, transcriptome analysis of FXS forebrain organoids revealed a significant number of genes with altered expression, whereas only a few were differentially expressed in the FXS mouse brain (Kang et al., 2021). Moreover, this analysis showed a large number of overlapped genes differentially expressed in FXS forebrain organoids and fetal brain tissues (Kang et al., 2021). The differentially expressed genes in the FXS forebrain organoids were associated with neuronal migration, axonogenesis, neurogenesis, and neuronal differentiation (Kang et al., 2021). In addition, the rescue of the developmental deficits in FXS forebrain organoids was achieved by inhibiting the PI3K pathway, but not the metabotropic pathway which was disrupted in the FXS mouse models (Guo et al., 2016), suggesting that brain organoids mimic more accurately the FXS phenotype (Kang et al., 2021). Although dysregulation of PI3K pathway has been linked with FXS pathogenesis (Gross et al., 2015), modulation of this pathway has not been performed in clinical trials yet. Moreover, considering that pharmacological inhibition of PI3K pathway could rescue some of the developmental defects in FXS forebrain organoids (Kang et al., 2021), using PI3K pathway as a target in future therapeutic strategies might be promising. Overall, all the above indicate that human brain organoids could serve as preclinical models to identify human-specific therapeutic targets (Figure 1B).

To investigate RTT, transgenic mouse models and post-mortem human tissues have been widely used. Both *Mecp2-*mutant mice and human patients exhibit motor abnormalities (Stearns et al., 2007), robust neurological symptoms including uncoordinated gait and reduced spontaneous movement (Guy et al., 2001), learning and memory deficits (Moretti et al., 2006), metabolic disturbances (Goffin and Zhou, 2012), increased oxidative stress (Janc et al., 2016), seizures, and a shortened lifespan (Stearns et al., 2007). However, the neurological symptoms in mice are less severe and do not fully recapitulate the human phenotype. Two-dimensional neuronal cultures differentiated from patient-derived iPSCs harboring *MeCP2* mutations showed impaired maturation, fewer synapses, excitatory/inhibitory (E/I) imbalance, smaller soma size and functional defects in firing activity (Marchetto et al., 2010). The recent innovations *o*f brain organ*o*ids were used for RTT modeling, revealing impaired neurogenesis in human MGEOs and cortical organoids (Xiang et al., 2020) as well as deficits in neurogenesis and neuronal differentiation in COs (Mellios et al., 2018). Lastly, assembly of dorsal (DFOs) and ventral forebrain organoids (VFOs) from RTT patient-derived iPSCs revealed defects in interneuron migration (Gomes et al., 2020), suggesting a valuable model for understanding RTT during early stages of neural development.

Rodent studies have shown that loss of *Tsc1/2* function in tuberous sclerosis impacts multiple processes at different developmental stages, including neuronal morphology and migration, synaptic plasticity and glial function (Tsai and Sahin, 2011). The germline loss of *Tsc1* in mice resulted in embryonic lethality prior to brain development (Kwiatkowski et al., 2002), thus, spatial and temporal genetic manipulation at very early stages of cortical neurodevelopment is necessary. Loss of Tsc1 in cortical neurons during embryonic development in mice led to several neurological abnormalities, ectopic enlarged neurons and seizure activity (Meikle et al., 2007). Nevertheless, although the conditional KO mouse models have been and will continue to be a powerful research tool, this pathology may result from unique cellular and molecular aspects of human brain development. Moreover, although one mutant allele of *TSC1/2* is sufficient to give rise to tuberous sclerosis in patients, heterozygous animal models demonstrate subtle or no symptoms (Goorden et al., 2007; Ehninger et al., 2008). Deficits in learning, memory and social behavior in *Tsc1*^+/–^ and *Tsc2*^+/–^ mice emerged in the absence of neuropathology and seizures (Goorden et al., 2007; Ehninger et al., 2008), indicating that animal models do not recapitulate faithfully the human phenotype. In contrast, the use of human stem cells enables to investigate the early stages of tuberous sclerosis neuropathology and identify the human-specific features of this disease (Figure 1B). It has been proposed that the haploinsufficiency of *TSC1/2* could engage the pathomechanisms responsible for the formation of cortical tubers, the neuropathological hallmark of tuberous sclerosis (Winden et al., 2019). Neurons differentiated from hESCs in 2D displayed altered synaptic transmission, which was rescued by inhibition of mTORC1 pathway (Costa et al., 2016). Moreover, 2D NPCs differentiated from patient-specific iPSCs exhibited increased size and enhanced proliferation, consistent with the mTORC1 signaling activation (Martin et al., 2020) as well as disrupted neuronal differentiation (Zucco et al., 2018). Likewise, 2D patient-derived primitive neural stem cells (pNSCs) displayed increased proliferative capacity while differentiated neurons exhibited enlargement of the soma, perturbed neurite outgrowth and disrupted connections with other cells (Li et al., 2017b). Interestingly, although patient-specific iPSC-derived *TSC2*^+/–^ neurons showed mTORC1 hyperactivation and associated increased cell body, only *TSC2^–/–^* neurons exhibited hyperactivity and transcriptional dysregulation observed in cortical tubers (Winden et al., 2019). A breakthrough in tuberous sclerosis research has been the development of patient-derived COs which recapitulated the emergence of brain tumors and dysplastic cortical regions (Eichmüller et al., 2022). More importantly, this model system revealed the presence of a novel interneuron progenitor population, namely CLIP cells, that give rise to both tumors and cortical tuber lesions (Eichmüller et al., 2022), highlighting the importance of using a proper model system in neurodevelopmental research.

#### Attention-deficit/hyperactivity disorder

Attention-deficit/hyperactivity disorder is a behavioral NDD characterized by ID, lack of attention, disorganization and difficulty in completing tasks (Ougrin et al., 2010). Although it is usually presented in children, symptoms might be maintained during adulthood. Findings from animal models suggest that ADHD is characterized by deficits in dopaminergic, noradrenergic and serotonergic systems, as well as more fundamental defects in neurotransmission (Russell, 2011). The etiology, although largely unknown, includes both genetic and environmental factors, while evidence shows that exposure to endocrine-disrupting chemicals, especially phthalates (Kim et al., 2009) and bisphenol A (BPA) (Rochester et al., 2018) might be causative. Genome-wide association studies have identified several candidate genes for ADHD including parkin 2 (PARK2) (Jarick et al., 2014), neuropeptide Y loci (Lesch et al., 2011) and genes in the metabotropic glutamate receptor family (GRM) (Elia et al., 2011). This polygenic etiology together with the variability in the clinical phenotype and the psychiatric root of ADHD renders its modeling extremely challenging.

Several animal models have been used to model ADHD including genetic, environmentally, and chemically induced models such as the spontaneously hypertensive rats (SHR) (Adriani et al., 2003), the Naples high-excitability rats (NHE) (Viggiano et al., 2002) and the neonatal 6-hydroxidopamine (6-OHDA) mice (Bouchatta et al., 2020). Since deregulation in the forebrain dopaminergic signaling and function has been associated with ADHD, dopamine transporter KO rodents (DAT-KO) have been generated by depleting *SLC6A3* gene to study ADHD. Indeed, the DAT-KO mice presented hyperactivity, sleep dysregulation and cognitive deficits (Kasahara et al., 2013) while the DAT-KO rats showed a deficit in working memory and behavioral alterations affecting reward processing and decision making (Cinque et al., 2018). Furthermore, ADHD mouse models suggested that environmental factors in prenatal and perinatal stages, including epigenetic modifications, premature birth, and maternal smoking during pregnancy are major risk factors for ADHD (Halmøy et al., 2009; Zhu et al., 2014).

Nevertheless, the limited knowledge that we have of the developmental origin of ADHD and the implicated molecular and cellular mechanisms led to the development of rapid and low-cost human-specific *in vitro* models. The generation of ADHD patient-derived iPSCs has been reported (Sochacki et al., 2016; Jansch et al., 2018), offering the unique potential to bridge the gap between genetic and neural network associations in ADHD in a human-specific environment. Screening of drugs and chemicals was achieved by developing brain organoids providing a cheaper, faster, and more biologically relevant model (Schwartz et al., 2015) in understanding the environmental predisposition in ADHD (Figure 1B). For instance, the study of the effect of BPA, which is known to affect also rodent neurodevelopment (Kundakovic et al., 2013), demonstrated that the forebrain organoid system allowed quantitative investigation of the consequences of BPA exposure (Qian et al., 2016). In addition, with the help of robotic technology, an automated approach of large-scale human midbrain organoid cultures was generated for screening the general and dopaminergic-specific toxic effects of several compounds (Renner et al., 2020). Nonetheless, the lack of the blood-brain barrier, from which many chemicals cross, may have an important effect on understanding the underlying mechanisms considering toxicity. Moreover, the lack of any behavioral readout in 2D/3D model systems is not negligible and should be taken into consideration. It is thus, commonly accepted that a single model cannot capture all the traits of a complex brain disorder and that a combination of different approaches is needed to fulfill this request (Figures 1A,B).

#### Other neurodevelopmental disorders

While the focus has been drawn on ASD and ADHD, especially in recent years, other NDDs such as ID, motor disorders, learning and speech disabilities are highly uncharacterized. Our knowledge regarding these NDDs has mainly derived from comparative studies in human subjects, as research in animal models is limited and 2D/3D *in vitro* studies have not been conducted yet. Considering that molecular genetic testing coupled with genome-wide association studies (GWAS) have identified causative genes in individuals with these NDDs (Niemi et al., 2018; Gialluisi et al., 2021; Mountford et al., 2021) it is of high importance to delve into the etiopathogenic mechanisms using both *in vivo* and *in vitro* research tools (Figures 1A,B).

### Malformations of cortical development characterized by the presence of ectopic neurons

#### Periventricular heterotopia

Individuals with periventricular heterotopia (PH) present a normal cortex with no major disruptions or changes. However, at the cellular level, patients exhibit clusters of gray matter organized either as laminar or as nodules close to the lateral ventricles. These areas of gray matter represent a subpopulation of neurons that fail to migrate toward the cortical plate (Watrin et al., 2015). Patients with PH suffer from various types of seizures and epilepsy, possibly due to the formation of an aberrant local neuronal network and synaptic connectivity. It has been proposed that the major etiopathogenic mechanisms of PH can be genetic mutations or environmental factors such as radiation, injury and infection (Watrin et al., 2015). While originally, PH was considered merely a neuronal migration disorder, recently focus has been drawn on the importance of NPCs’ proliferation and differentiation in the disease manifestation.

The X-linked *FLNA* which stabilizes the cytoskeleton was one of the first genes that have been associated with PH using animal models. The *Flna*-knockdown (KD) mouse model showed the importance of radial glia organization and the function of progenitor cells during neurogenesis and migration (Carabalona et al., 2012). Likewise, a PH ferret model exhibited neuronal nodules located in the cortex and disorganized radial glial fibers (Matsumoto et al., 2017). The significance of microtubule and actin dynamics in PH pathogenesis was also highlighted with the identification of *MAP1B*, encoding the microtubule-associated protein 1B, as a risk factor for PH (Heinzen et al., 2018). Interestingly, while pathogenic mutations in the microtubule-associated protein EML1, leads to subcortical band heterotopia in humans (Shaheen et al., 2017) (Section “Subcortical band heterotopia”), disruption of the Eml1 resulted in the heterotopic cortex (HeCo) in mouse mutants (Kielar et al., 2014). HeCo mice exhibited a population of aRGs located away from the VZ, which depicted perturbed primary cilia and disrupted Golgi apparatus function (Bizzotto et al., 2017; Uzquiano et al., 2019). In addition, Cappello et al., using also the mouse model demonstrated that Fat4-Dchs1 and Yap are key regulators in mammalian neurogenesis, suggesting that changes in the number of progenitor cells and their differentiation ability led to heterotopic neurons in the subcortex (Cappello et al., 2013). Notably, a Hippo signaling factor serving as a molecular modulator of Fat4 and Dchs1, namely Mob2, has also been implicated in PH pathogenesis (O’Neill et al., 2018a). *Mob2* KD in the developing mouse cortex altered neuronal distribution and led to impaired ciliary numbers and position within migrating neurons (O’Neill et al., 2018a). Mechanistically, it has been also proposed that vesicle and/or membrane trafficking from the trans-Golgi network changes the transport of polarized molecules to the radial glial cells’ surface, thereby disrupting their proliferation and migration during cortical development (Sheen, 2012). For instance, mutations in the *ARFGEF2* gene encoding the vesicle trafficking Brefeldin A-associated guanine exchange factor 2 (BIG2), are implicated in the manifestation of PH in humans (Sheen, 2014). Moreover, disruption of the E3 ubiquitin ligase NEDD4L leads to PH in humans, with mutants showing sensitivity to proteasome degradation in mouse models (Broix et al., 2016). Mutations in *INTS8* have been also linked with severe NDDs characterized by PH, profound cognitive impairment, limb and facial dysmorphism and epilepsy, with patients exhibiting global transcriptome perturbations (Oegema et al., 2017).

Even though extensive research has been done in the past years using animal models, these systems failed to faithfully recapitulate the human phenotype. More recent work in 2D and 3D human-specific model systems highlighted the importance of the morphology and function of NPCs in the manifestation of PH (Klaus et al., 2019). Specifically, patient-derived *DCHS1* and *FAT4* deficient COs presented numerous neuronal nodules at ventricular positions, poorly organized germinal zones and morphologically disrupted NPCs. Additionally, KD of these genes in control COs led to an aberrant migration pattern in a subset of neurons (Klaus et al., 2019). Interestingly, single-cell RNA-sequencing analysis revealed a subpopulation of neurons with dysregulated genes involved in axon guidance, neuronal migration and patterning, elucidating the phenotype observed in patients, where only a subgroup of neurons fail to migrate, generating ectopic nodules (Klaus et al., 2019). Among the differentially expressed genes in the patient-specific ectopic cluster of neurons, *GNG5*, which is naturally expressed in NPCs, was the most upregulated (Klaus et al., 2019), indicating that its downregulation is essential for proper neuronal migration. Overexpression of *GNG5* in control COs provoked premature NPCs’ delamination, induced alterations in NPCs’ morphology and led to neuronal migration defects (Ayo-Martin et al., 2020). Hence, it is now believed that the cross-talk between NPCs’ morphology and altered migration behavior of newly born neurons is a human-specific molecular and cellular explanation for PH manifestation (Klaus et al., 2019; Ayo-Martin et al., 2020). The importance of a human-specific model to study PH was also highlighted in a publication describing a novel *de novo* mutation in the human-specific isoform of the *PLEKHG6*, which is responsible for altering progenitors’ differentiation and neuronal migration downstream of RhoA (O’Neill et al., 2018b) highlighting again the necessity for the use of human-specific model systems. Besides, by using brain organoids, cell non-autonomous mechanisms have also been implicated in the etiology of PH by modeling the role of *ECE2* (Buchsbaum et al., 2020), demonstrating the importance of the extracellular organization in the manifestation of PH. Likewise, mutations in *LGALS3BP*, encoding a secreted protein that interacts with the ECM, have been associated with PH, autism and seizures (O’Neill et al., 2018b). *LGALS3BP* deficiency led to increased apical belt thickness, prevention of NPCs’ delamination, ectopically located neurons and decreased RGs’ ciliary length in COs, indicating that its expression affects corticogenesis (Kyrousi et al., 2021). This study suggested that ECM, with LGALS3BP being a mediator of ECM signals, is crucial for human cortical development at the cellular level. Interestingly, patients with *LGALS3BP* mutations exhibit also changes in the local gyrification index, characteristics of other MCDs (Section “Malformations of cortical development characterized by altered gyrification index”), reinforcing the comorbidity between different MCDs.

#### Subcortical band heterotopia

Subcortical band heterotopia (SBH) is characterized by a smooth band of gray matter in the superficial and middle portions of the white matter. It emerges when newly generated neurons fail to migrate to the correct location and accumulate in the white matter. In humans, SBH is associated with ID, drug-resistant seizures and epilepsy (Sahu et al., 2019). The majority of patients are females carrying mutations in the X-linked *DCX* gene, exhibiting diffuse thick bands of ectopic neurons (Dobyns, 2010). Due to the stochastic nature of X-inactivation females with *DCX* mutations present a mosaic state with two populations of neurons: one that expresses DCX and migrates properly and the deficient one that gives rise to the heterotopic band. In males with DCX deficiency neurons lack completely the protein, giving rise to the more severe lissencephalic “smooth” cortex (Section “Lissencephaly”) (Matsumoto et al., 2001). Another locus that has been linked with SBH contains the *PAFAH1B1* gene, which encodes the LIS1 protein. *LIS1*-associated SBH exhibits thin or intermediate posterior bands, with posterior heterotopia being more severe than anterior heterotopia (Mineyko et al., 2010). Additionally, individuals with mutations in *EML1*, exhibit SBH, megalencephaly, polymicrogyria (section “Polymicrogyria”), and agenesis of corpus callosum (Shaheen et al., 2017; Oegema et al., 2019). Overall, MCDs result from single point mutations in critical developmental genes or from variations in several genetic loci, increasing disease probability. Interestingly, disruption of common genes might lead to divergent disease phenotypes (Table 1), with the variable penetrance of genetic variants complexifying, even more, the overall clinical manifestation (Klingler et al., 2021).

Although KD of *Dcx* in rats has provided valuable insight into the SBH epileptogenicity, they only harbor a unilateral SBH, not mimicking accurately the human phenotype (Lapray et al., 2010). Disruption of *Eml1* leads to HeCo mutant mice, a unique model that has been widely used to decipher the molecular basis of SBH. HeCo mice exhibit progenitor anomalies with some RGs leaving the VZ and diving in basal locations (Kielar et al., 2014). HeCo mice RGs presented disrupted primary cilia and perturbed Golgi apparatus mechanisms that probably contributed to the abnormal RGs’ delamination (Bizzotto et al., 2017; Uzquiano et al., 2019). The contribution of primary cilia in SBH pathophysiology is not surprising considering their role in the regulation of RG behavior. Nonetheless, although HeCo mouse models exhibit heterotopia they do not present the whole spectrum of pathologies found in humans, such as polymicrogyria and megalencephaly, indicating differential disease manifestation in humans and non-human models.

The emergence of cutting-edge stem cell technology has been extremely valuable in deciphering the etiopathogenesis of SBH. Two-dimensional cortical progenitors differentiated from *EML1* mutant iPSCs exhibited perturbed primary cilia formation and disrupted function of Golgi apparatus (Uzquiano et al., 2019). Moreover, *EML1*-deficient forebrain organoids presented ectopic neural rosettes accumulated at the basal side of VZ and heterotopic neuronal clusters (Jabali et al., 2022). RGs in the VZ of mutant organoids exhibited altered spindle orientation and shorter primary cilia which might directly impact RG delamination. Additionally, single-cell RNA-sequencing in the ectopic progenitor populations showed upregulation of bRGs markers and human-specific ECM components (Jabali et al., 2022). Although this model system revealed the implicated pathomechanisms in EML1 deficiency, the developmental stage of organoids does not allow us to study the full scope of MCDs caused by *EML1* impairment.

### Malformations of cortical development characterized by altered gyrification index

#### Lissencephaly

Lissencephaly is characterized by defective neuronal migration and results in the lack of development of sulci and gyri which leads to a smooth brain surface. It encompasses a broad group of brain malformations with abnormalities in the formation of gyri ranging from agyria (absence of gyri) to oligogyria (reduced gyrification) (Severino et al., 2020). Besides those, pachygyria (broad gyri) and SBH (double cortex) are also included in the spectrum of Lissencephaly (Kattuoa and Das, 2022). Depending on the degree of malformation, patients might present developmental delay and mental disabilities. Among the key mechanisms leading to Lissencephaly is the disruption of the proper development of gyri and sulci. It is caused due to genetic and non-genetic factors such as lack of oxygenated blood to the brain during fetal development or viral infections of the mother of the fetus (Leruez-Ville and Ville, 2017). Interestingly, many associated genes are causative for the manifestation of SBH as well (section “Subcortical band heterotopia”), supporting the notion that impairments in common genes result in divergent clinical pictures. Miller Dicker Syndrome (MDS) is a cortical malformation characterized by Lissencephaly often associated with microcephaly (section “Microcephaly”) leading to ID and mortality (Herman and Siegel, 2008). This syndrome is caused by deletions in the human band 17p13.3, which includes *PAFAH1B1* (LIS1 protein) (Herman and Siegel, 2008). Genetic causes of Lissencephaly also involve the *DCX* gene which is essential for proper neuronal migration (Corbo et al., 2002). Other genetic causes of Lissencephaly include mutations in *ARX* playing an important role in forebrain development (Gécz et al., 2006), *RELN* which encodes the protein reelin (Hong et al., 2000) and other genes like *KIF2A, KIF5C, CDK5, VLDLR, ACTB, ACTG1*, and *TUBG1* (Parrini et al., 2016) which, however, have not been properly modeled so far.

The function of Lis1 and the pathways associated with it have been studied in mouse models for Lissencephaly (Youn et al., 2009). Mice with a graded reduction in Lis1 dosage in disorganized cortical layers, cerebellum, hippocampus and olfactory bulb have been suitable models to study MDS. These models have aided in demonstrating impairments of motor coordination and cognition, severe disruptions of hippocampal cellular and synaptic physiology as well as *in vivo* migration defects (Paylor et al., 1999; Fleck et al., 2000). Additionally, NDEL1 which interacts with the LIS1/dynein complex has also been implicated in Lissencephaly pathogenesis. Specifically, *Ndel1* null mouse brains exhibited severe cortical layering defects and hippocampal deficits (Youn et al., 2009). Another genetic cause associated with classic Lissencephaly, and SBH is mutations in the *DCX* gene. *Dcx* mutant mice displayed disrupted lamination in the hippocampus, leading to deficits in learning (Corbo et al., 2002). However, the small and unfolded smooth brain of mice makes it difficult to model the wide spectrum of phenotypes in a precise way. For example, despite the disruption of the hippocampus, *Dcx* mutant mice presented a normal cortex with regular patterns of neocortical neurogenesis and neuronal migration (Corbo et al., 2002). In contrast, small interfering RNA (siRNA) inhibition of *DCX* in rat embryonic cortical slices led to severe defects in neuronal migration (Bai et al., 2003). These opposing results suggest that there is a more complex role for *DCX*, highlighting the necessity of human-specific models.

Differentiation of iPSCs into 2D neural cells provides a model system to further scrutinize the molecular and cellular regulation of Lissencephaly and in particular, the function of *DCX* in neurodevelopment. Neural stem cells (NCSs) with absent or reduced DCX protein expression showed delayed differentiation, impaired migration and neurite formation, recapitulating the lissencephalic phenotype (Shahsavani et al., 2018). As previously mentioned, in the developing human cortex the oSVZ, in which IP cells and bRGs are localized, is thicker compared to other mammals (Hansen et al., 2010). While IPs are conserved between human and mouse, bRGs are fewer in the developing cortex of lissencephalic mice. Moreover, human bRGs have been associated with the evolutionary increase in the cortical size of the human brain (Nonaka-Kinoshita et al., 2013). All the above may explain why Lis1 deficient mice presented a milder phenotype compared to human patients with heterozygous *PAFAH1B1* mutations. The implementation of brain organoid cultures has demonstrated the effect of MDS Lissencephaly mutations on distinct biological processes and cell types. Specifically, there has been a recapitulation of developmental lineage from NEs to RGs, IPs and bRGs with spatial and temporal resolution, which could not have been achieved with the previous methods (Kadoshima et al., 2013). This has highlighted the vulnerability of NEs and human-specific bRGs in MDS, whereas aRGs and IPs were less affected (Bershteyn et al., 2017). MDS-forebrain organoids exhibited small size, reduced expansion rate, changes in RGs’ division plane, increased neurogenesis and altered cortical niche architecture (Iefremova et al., 2017). Finally, the involvement of the ECM in cortical gyrification and thereby, in the manifestation of MCDs characterized by altered gyrification index is not insignificant considering that patients harboring *LGALS3BP* mutations, exhibit altered gyrification, while its overexpression in the mouse brain promoted cortical folding, indicating that LGALS3BP regulates cortical expansion and gyrification (Kyrousi et al., 2021). Even though bRGs seem to have a robust effect on gyrification and hence, the emergence of MCDs with altered gyrification index, due to the almost total absence of bRGs in mice, it is challenging to study bRG behavior in this model. In contrast, in brain organoids, there are bRG-like cells with matching characteristics *in vivo* such as morphology, molecular identity and mitotic behavior. Nevertheless, how the *in vitro*-derived bRG cells recapitulate the functional properties and molecular identity of primary bRG cells has not yet been determined, making the synergy of *in vivo* and *in vitro* models essential (Figures 1A,B).

#### Polymicrogyria

Polymicrogyria (PMG) is a MCD characterized by the presence of multiple abnormally small gyri which leads to an irregularly folded cortical surface with an increased gyrification index. Individuals with PMG present cognitive impairments, epilepsy and developmental delay. It has been associated with the malfunction of several genes including *TUBA1A* (Cushion et al., 2013), *TUBB2B* (Guerrini et al., 2012), *TUBB3* (Poirier et al., 2010), *GPR56* (Piao et al., 2004), *WDR62* (Yu et al., 2010), *EOMES* (Baala et al., 2007), *SCN3A* (Smith et al., 2018), as well as environmental causes. Considering that bRGs are responsible for cortical folding in gyrencephalic species, PMG might be an outcome of increased migration and/or excess of bRGs (Klingler et al., 2021). It is a highly heterogeneous disorder in terms of topographic distribution, clinical, and imaging features, as well as severity levels, varying from focal forms to bilateral involvement (Leventer et al., 2010). PMG often occurs concurrently with other MCDs, such as Lissencephaly, macrocephaly and heterotopia (Stutterd et al., 2020), but it is probably the least characterized among the more common MCDs (Stutterd and Leventer, 2014), thus more effort has to be made to identify the implicated mechanisms.

Only a few animal models harboring mutations in PMG-associated genes, that are mentioned here, have been reported (Table 1). Nonetheless, they fail to recapitulate accurately the human phenotype. For instance, *Tubb2-*deficient mouse model exhibited cortical thinning and increased apoptosis of cortical neurons which led to perinatal lethality (Stottmann et al., 2013). Likewise, while individuals carrying *EML1* mutations exhibit a wide spectrum of impairments including a PMG-like cortex, HeCo mouse models failed to mimic this phenotype (Kielar et al., 2014) (section “Subcortical band heterotopia”). Additionally, although loss of GPR56 in humans leads to bilateral frontoparietal PMG (Piao et al., 2004), in mice it led to ectopic cortical neurons due to neuronal over-migration, structural aberrations in the RG endfeet and breaches in the pial basement membrane (Li et al., 2008). Overall, Gpr56 mutant mice exhibited disorganized cortical lamination and a cobblestone-like malformation (Li et al., 2008). Although mouse models have been a valuable tool in MCDs research, using complex gyrencephalic cortices is essential to decipher the PMG-implicated pathomechanisms. Interestingly, the PMG-associated *GPR56* e1m promoter preferentially drove gene expression in GABAergic neurons of the developing cortex in common marmosets, indicating a possible link with *GPR56*-mutation associated epilepsy (Murayama et al., 2020). Moreover, altered expression of *SCN3A*, which encodes the brain-enriched voltage-gated sodium channel Na_*V*_1.3, disrupted cortical gyral formation and led to cortical gray matter heterotopia in a ferret animal model, highlighting the necessity of ion channels function at early stages of cortical development (Smith et al., 2018).

Even though initial steps have been taken to model PMG, animal models cannot mimic properly the human clinical phenotype, therefore, the need for human-specific model systems is urgent. Additionally, the lack of human-specific models for several PMG-linked genes, such as *GPR56*, impedes the disease characterization and modeling, hence steps need to be taken toward this direction. Overexpression of *SCN3A* mutant variants in 2D primary human neuronal cultures attenuated neurite branching, supporting the notion that *SCN3A* regulates neuronal development (Smith et al., 2018). Transcriptome analysis in *EML1*-deficient COs revealed an upregulation of bRGs markers and human-specific ECM components in the ectopic neural rosette population, supporting the involvement of bRGs in PMG pathophysiology (Jabali et al., 2022). Considering that the upregulated ECM genes *COL1A2, COL3A1*, and *LUM* have been linked with cortical expansion and folding (Long et al., 2018), it is tempting to speculate that the increase in their activity plays a role in the development of PMG-like phenotype in patients harboring *EML1* mutations (Oegema et al., 2019). Moreover, modeling the WDR62-associated microcephaly gene (section “Microcephaly”) using COs depicted disruption of bRGs (Zhang et al., 2019), indicating that this model system could be a valuable tool to delve into PMG implicated pathways. Nonetheless, the absence of folding in brain organoids constrains the modeling of PMG-associated phenotypes. Deletion of PTEN in human COs led to the expansion of the NPC population and substantial surface folding (Li et al., 2017c), indicating that we are one step closer to engineering folded brain organoids that can be used for PMG modeling. While actions have been taken toward the generation of folded organoids, the existing protocols are preliminary. In contrast to cortical gyrification which takes place around gestational week 30, when the fetal brain is largely composed of neurons, folded organoids contain mainly NPCs, thus they mimic an earlier developmental stage (Karzbrun et al., 2018). Overall, while the organoid wrinkling is equivalent to cortical gyrification there are crucial biological differences, indicating that this field is still developing.

### Malformations of cortical development characterized by abnormal brain size

#### Microcephaly

Microcephaly is characterized by reduced brain growth and is defined as a head circumference with ≤−3 SD. It results from an imbalance between progenitors’ production and cell death and leads to a reduced number of neuronal and glial cells (Ashwal et al., 2009; Álamo-Junquera et al., 2015). It is one of the most studied MCDs, with extensive research showing that some of the causes of primary microcephaly (present at birth) are impairments in DNA repair pathways (Zhou et al., 2013), mutations in genes encoding centrosomal proteins (Guernsey et al., 2010), double-stranded breaks (Yue et al., 2013), toxic exposures (Parens et al., 2017), *in utero* infection (Antoniou et al., 2020) and metabolic conditions (Ashwal et al., 2009; Table 1). Secondary microcephaly (postnatal manifestation) can be associated with migration defects (Juric-Sekhar et al., 2011), increased cell death (Simmons et al., 2019), and metabolic disorders (Ashwal et al., 2009). Besides, microcephaly can also be a clinical feature in other NDDs and syndromes like RTT (Banerjee et al., 2012), Angelman syndrome (Sun et al., 2019) and Seckel syndrome (Al-Dosari et al., 2010), while, maternal infections from Zika virus (ZIKV) lead to congenital zika syndrome (CZS) with infants exhibiting severe microcephaly (Moore et al., 2017; Antoniou et al., 2020).

Zebrafish has been a useful animal model to decipher the genetic implications and molecular mechanisms of microcephaly. Knocking down of the abnormal spindle-like microcephaly associated gene (*Aspm*), which is associated with the manifestation of autosomal recessive primary microcephaly (MCPH), resulted in reduced head size in zebrafish (Kim et al., 2011). Zebrafish *Fnacd2* mutants also presented microcephaly due to increased p53-dependent apoptosis (Liu et al., 2003). Moreover, immunocompromised (AG129, C57BL/6 models) (Shao et al., 2016; Julander et al., 2018; Li C. et al., 2018) and immunocompetent [Swiss Jim Lambert (SJL), ICR, STAT2 KO models] (Li et al., 2016; Gorman et al., 2018; Szaba et al., 2018) mice have been a useful tool to investigate the effects of ZIKV infection. In addition, mouse models have been widely used to investigate the effects of disruption of MCPH-associated genes in the developing brain (McIntyre et al., 2012; Insolera et al., 2014; Zhang et al., 2019). Loss of CPAP, a protein crucial for centriole biogenesis, induced p53-dependent cell death which severely disrupted embryonic mouse brains (Lin et al., 2020). However, patient mutations in mice led to mild microcephalic phenotypes and did not give insights into cellular and molecular mechanisms. Additionally, there is no clear evidence that a disease-causing mutation in the mouse brain is analogous to human microcephaly. Interestingly, *Aspm* KO in the ferret, a species with a larger gyrencephalic cortex and greater NPC diversity than mice, led to severe microcephaly with significantly reduced cortical surface area (Johnson et al., 2018). To recapitulate more accurately the pathogenesis, neurophysiology and therapeutic development of CZS, NHPs have also been used as they present similarities to humans in gestational biology (Nguyen et al., 2017). Specifically, macaques recapitulated key features of human microcephaly via ZIKV infection (Dudley et al., 2019). Nevertheless, the demands of NHS breeding and management as well as the generation of transgenic animals are challenging, highlighting the need for model systems that are not only physiologically relevant to the human brain but also easy to manipulate.

Human-specific 2D and 3D *in vitro* models have contributed to decoding the complexity of microcephaly, which was challenging to be deciphered in the existing *in vivo* models. Differentiation of Seckel patient-derived iPSCs in 2D, revealed reduced proliferation and elongated cilia in *CPAP* mutant NPCs (Gabriel et al., 2016). Interestingly, Seckel NPCs exhibited retarded cilium disassembly and delayed G1-S transition, which led to NPC premature differentiation (Gabriel et al., 2016). In the same line, 2D differentiation of hESCs into NPCs harboring mutations in the microcephaly associated gene *KNL1*, which is essential for spindle assembly checkpoint signaling, revealed reduced cell growth, high cell death, and premature differentiation at the expense of NPC proliferation (Omer Javed et al., 2018). *CDK5RAP2*, which encodes the CDK5 regulatory subunit-associated protein 2, was the first gene used to study microcephaly in brain organoids. The generation of patient-derived COs has shown that changes in the mitotic spindle orientation affect progenitors’ proliferation and lead to premature neuronal differentiation (Lancaster et al., 2013). Moreover, modeling *ASPM* mutations demonstrated the ability of human COs to recapitulate microcephaly (Li et al., 2017a). In turn, *CPAP* mutant COs displayed reduced size—a key feature of microcephaly—and increased number of cilia which affected progenitors’ mode of division and led to premature neurogenesis (Gabriel et al., 2016). Likewise, a *CPAP* mutation identified in MCPH patients induced cell death in COs and led to the generation of smaller organoids (An et al., 2022). Additionally, the *CPAP* mutation provoked NPC spindle misorientation which resulted in premature differentiation (An et al., 2022). Finally, *WDR62* deletion, which encodes a centrosomal protein, in COs revealed impaired ciliary length, decreased NPC proliferation and premature neuronal differentiation which eventually led to a reduction in the size of mutant organoids (Zhang et al., 2019). The perturbed ciliary structure and function in the 2D/3D models of microcephaly highlight the necessity of primary cilia in regulating crucial developmental processes including NPCs’ proliferation and differentiation. Last but not least, brain organoids have successfully recapitulated the effects of ZIKV infection in cortical development, revealing a RG-specific infection, especially in the VZ, resulting in a reduction of the proliferative zones (Cugola et al., 2016).

#### Macrocephaly

Macrocephaly is defined as head circumference with ≥2 SD for a given age and gender (Accogli et al., 2022), originating from a higher proliferation rate of progenitors as well as defects in growth factor signaling (Sun and Hevner, 2014). Heterozygous loss-of-function mutations in *PTEN* were identified in patients with macrocephaly and associated with ASD, concurrently (Butler et al., 2005). Individuals with *PTEN* mutations exhibit brain enlargement and white matter abnormalities (Vanderver et al., 2014). Nonetheless, *PTEN* mutations do not have the same effects throughout different brain areas. Abnormal scaling of different brain regions could indicate disrupted connectivity, the presence of ectopic tissue, or abnormal innervation of crucial brain areas, which may contribute to behavioral phenotypes. Interestingly, loss of *PTEN* leads to an increase in the PI3K-AKT-mTOR signaling, which in turn has been suggested to have important roles in macrocephaly (Yeung et al., 2017). For instance, mutations in the small GTPase RAB39B which interacts with the PI3K components are associated with X-linked macrocephaly, ASD and ID (Woodbury-Smith et al., 2017). Moreover, *AKT3* activating mutations have been linked with macrocephaly and polymicrogyria (Lee et al., 2012).

Prenatal neurodevelopmental abnormalities in macrocephaly are difficult to be understood because of the limited access to human brain tissues. Thus, genetically modified animal models remain a necessary strategy to scrutinize the pathogenesis of macrocephaly on a molecular and behavioral basis. *Rab39b* KO mice exhibited macrocephaly and autism-like behaviors (Zhang et al., 2020). In particular, they presented an increased proliferative rate and decreased cell cycle exit of NPCs which led to the expansion of the progenitors’ pool (Zhang et al., 2020). *Pten*^+/–^ mouse models presented regional brain overgrowth, which is similar to brain enlargement in adult *PTEN*-ASD patients (Clipperton-Allen et al., 2019). This phenotype may be associated with increased gliogenesis due to reduced suppression of the PI3K pathway (Clipperton-Allen et al., 2019). In addition, *Akt3* mutant mice harboring a dominant missense mutation showed enhanced signaling activity and increased brain size (Tokuda et al., 2011).

In humans, the majority of upper layer neurons derive from the oSVZ, where bRGs are abundant. On the contrary, in rodents, bRG-like cells are fewer in number. Thus, human iPSCs and iPSC-derived brain organoids are a valuable platform for studying macrocephaly, as the human brain exhibit increased cortical size and complexity. Indeed, *RAB39b* mutant COs exhibited a more severe phenotype (Zhang et al., 2020) compared to KO mouse models, highlighting the valuable role of brain organoids in modeling human early development. These mutant brain organoids had a more prominent increase in the neural progenitor pool and upper layer neurons compared to mutant mice, explaining the severe phenotype that appeared in mutant organoids (Zhang et al., 2020). Likewise, the deletion of *PTEN* resulted in enlarged and folded brain organoids. *PTEN* homozygous mutations in organoids increased PI3K pathway signaling, promoted cell cycle re-entry of NPCs and transiently delayed neuronal differentiation, resulting in expansion of the progenitor population (Li et al., 2017c). However, the fact that the developmental timing between organoids and mutant mice is not compatible, shows the importance of making side-by-side comparisons between mouse models and human brain organoids.

## Brain organoids as tool to model neurodegenerative and neuropsychiatric disorders

Besides the applications of brain organoids in NDDs research, they can be also used to elucidate impaired processes in neurodegenerative and neuropsychiatric diseases (Sun et al., 2021). The poor physiological relevance of animal models’ brain, the limited number of therapies as well as the inefficient treatment effects in neurodegenerative disorders dictate the need for revolutionary tools. Moreover, the requirement for a non-invasive analysis of patient-derived tissue and an effective drug-screening system has contributed to the emergence of brain organoid cultures. Even though brain organoids do not recapitulate the aging brain, they have been used to model several neurodegenerative disorders. Since these disorders are associated with extended periods of prodromal disease, where cellular abnormalities are accumulating without manifesting as clinical symptoms, organoids allow us to investigate these early cellular changes that drive neurodegeneration (Wray, 2021). Moreover, advances in organoid technology have enabled the protracted culture of brain organoids, leading to increased cellular diversity and neuronal maturation (Quadrato et al., 2017). These matured organoids developed spontaneously active neuronal networks and acquired traits of mature neurons, suggesting that, beyond modeling early neurodevelopmental processes, they have the potential to model higher-order functions of the human brain (Quadrato et al., 2017). Organoids have been used to model Alzheimer’s disease (AD) (Gonzalez et al., 2018; Ghatak et al., 2019), Parkinson’s disease (PD) (Kim et al., 2019; Smits et al., 2019), amyotrophic lateral sclerosis (ALS) (Osaki et al., 2018), frontotemporal dementia (Seo et al., 2017), Huntington’s disease (Conforti et al., 2018), and spinal muscular atrophy (Hor et al., 2018). Considering that 2D model systems lack the complex extracellular environment necessary to recapitulate protein aggregation, such as the extracellular deposition of misfolded amyloid-β (Aβ) plaques, the molecular hallmark of AD, 3D culture conditions provide a powerful tool to investigate Aβ pathology (Lee et al., 2016; Bubnys and Tsai, 2022). Additionally, the emergence of human midbrain organoids has been pioneering in PD research, which is characterized by the loss of dopaminergic neurons in the substantia nigra pars compacta and the presence of Lewy body inclusions (Galet et al., 2020). Likewise, the complexity of the human brain concerning physiological, functional and structural aspects of brain development as well as the increase in cognitive abilities have pointed out the need for human-specific model systems to study neuropsychiatric diseases. Toward this direction, organoids have provided valuable information regarding implicated pathomechanisms in schizophrenia (Kathuria et al., 2020), bipolar disorder (Mertens et al., 2015), psychosis (Sawada et al., 2020), and depression (Vadodaria et al., 2019). In particular, modeling the effect of schizophrenia-linked genes using brain organoids revealed disrupted neural rosette structures and altered proliferation of NPCs which was rescued by WNT antagonism, indicating that organoids can be a valuable tool for drug testing (Figure 1B) (Srikanth et al., 2018). Taking into account, that patients with NDDs can exhibit simultaneously neurodegenerative or neuropsychiatric disorders, the use of a model system that can recapitulate them faithfully is of great potential.

## Conclusion and future perspectives

Creating a functional cerebral cortex requires the coordination of crucial developmental processes such as progenitors’ proliferation and differentiation, neuronal migration and eventually their maturation and integration into the existing neuronal network. Disruptions of these complex and delicate processes lead to a plethora of brain impairments, the NDDs. Their clinical manifestation encompasses developmental delay, epilepsy, ID but also various structural abnormalities that affect the size, thickness and folding of the cortex. Understanding the molecular etiology of NDDs is crucial for patient management. Nonetheless, their multifactorial and polygenic nature makes their modeling difficult. Although rodent and other animal models have been the foundation for deciphering the implicated pathways in NDDs, the use of human-specific models is essential. Toward this direction, iPSC-derived 2D and 3D *in vitro* cultures show great potential for modeling human neurodevelopmental pathologies. In this review, we elaborated on both the *in vivo* and *in vitro* models used to study NDDs (Figures 1A,B).

Although the tremendous progress in stem cell and organoid development has decoded complex pathogenic pathways, brain organoids present numerous limitations as a model system (Paşca, 2018). In particular, they do not develop past the stage of a prenatal brain and cannot recapitulate the six-layered spatial organization of the cortical plate (Lancaster et al., 2013). Nevertheless, recent advances in the patterning of brain organoids have led to prolonged *in vitro* development, enabling significant levels of maturity (Quadrato et al., 2017; Velasco et al., 2019). Additionally, despite the existence of bRGs, COs have not presented folding on the pial surface and gyrification (Qian et al., 2019). Moreover, the E/I neurons although functional, are less mature compared to adult neurons. Thus, cell diversity in organoids remains to be further enriched, also given that they lack non-neuronal cell types such as microglia (Dutta et al., 2017) which could be achieved with new pattering protocols (Ormel et al., 2018) or with co-cultures with non-neuronal lineage cell types in the future. The lack of vascularization, which restricts the delivery of nutrients and oxygen has been another significant obstacle to the long survival of these cultures, as a necrotic core is built up inside the organoids (Qian et al., 2019). Toward addressing the lack of vascularization, the grafting of human brain organoids into adult mouse brains has been performed (Mansour et al., 2018). Interestingly, functional intra-graft neuronal networks, as well as graft-to-host synaptic connectivity were observed in these mice. Another promising tool for overcoming such problems is organoids-on-a-chip. A recent report on the human kidney has examined the fluid flow on vascularization and maturation of hiPSC-derived kidney organoids. They engineered them in a 3D printed chamber which led to the expansion of endothelial progenitors within the organoids and eventually the formation of blood vessels in a flow-enhanced way (Homan et al., 2019). Another limitation is the variability of the different organoid cultures, which depends on the quality of the founder cell line (i.e., the line and passage of the iPSCs) (Hernández et al., 2022). Lastly, one major obstacle to the use of brain organoids to model NDDs is the lack of any behavioral readout of the *in vitro* cultures. Summarizing, focusing on six-layer human architecture, *in vitro* vascularization, patterning and further refinement in tissue identity can offer great promise, toward the development of a more robust human-specific model system (Chiaradia and Lancaster, 2020). Nonetheless, the advanced development and engraftment of organoids in animal brains constitute an ethical gray zone, making ethicists wonder if these experiments are essential to answer a scientific question or whether boundaries are pushed beyond limits (Reardon, 2020; Powell, 2022).

## Author contributions

ED and LM wrote the manuscript. CK conceived, wrote, and revised the presented work. All authors read and approved the final manuscript.
